# Comparison of two different methods of fiber-optic nasal intubation: conventional method versus facilitated method (NASAL-18)

**DOI:** 10.3109/03009734.2010.534827

**Published:** 2011-04-12

**Authors:** Ali Mohammadzadeh, Mohammad Haghighi, Bahram Naderi, Amanullah Chaudhry, Mohammad R. Rasouli, Soheil Saadat, Zahid Hussain Khan

**Affiliations:** ^1^Department of Anesthesiology, Guilan University of Medical Sciences, Rasht, Iran; ^2^Department of Anesthesiology, Razi Hospital, Rasht, Iran; ^3^Department of Anesthesiology, Imam Khomeini Medical Center, Tehran University of Medical Sciences, Tehran, Iran; ^4^Sina Trauma and Surgery Research Center, Tehran University of Medical Sciences, Tehran, Iran

**Keywords:** Facilitating method, fiber-optic intubation, nasal intubation

## Abstract

**Objectives:**

As the conventional fiber-optic nasal intubation technique has several potential difficulties, we compared in this study another technique (NASAL-18) with the conventional one in attempting fiber-optic nasal intubation with a possible higher rate of success.

**Methods:**

A randomized controlled trial was carried out at a teaching hospital. Forty-eight patients aged 25–45 years with American Society of Anesthesiologists (ASA) classes I and II undergoing elective faciomaxillary surgery were allocated to two groups of fiberscopic nasal intubation using either the NASAL-18 technique or the conventional method (control). In the NASAL-18 group, a nasal tube was gently inserted into the nasopharynx till mark 18, then a fiberscope with 41 cm length was glided over it and advanced through the nasal cavity till the glottis could be visualized. Finally the nasal tube was rolled over the fiberscope instead of one-step passage of the nasal tube after visualization of glottis in the controls. Times from the start of insertion of the fiberscope into nares till visualization of vocal cord (T1) and from here to complete intubation (T2) were recorded. These times were compared between the two groups.

**Results:**

T1 values in NASAL-18 and control groups were 65.2 ± 33.2 and 151.0 ± 56.5 seconds, respectively (*P* < 0.0001). T2 durations were measured as 25.1 ± 18.5 and 21.8 ± 10.1 seconds in control and NASAL-18 groups, respectively (*P* = 0.45). The NASAL-18 group had a success rate of 83% compared to 66.7% in the control group.

**Conclusions:**

The NASAL-18 method reduces the time needed for successful fiber-optic intubation. This method can be added to the list of techniques in facilitating fiber-optic intubation.

## Introduction

Operations that are conducted on or in the vicinity of the jaws, in which the mouth is being used for manipulation or insertion of devices, and where there is limited jaw mobility, make usage of a conventional laryngoscope extremely difficult or even impossible (1–4). Even today, years after the first application of the fiberscope, anesthesiologists still encounter difficulty in intubation in 90% of cases (4–7).

The conventional fiber-optic nasal intubation method in which the nasal tube is inserted in one step after visualization of the vocal cords by a flexible fiber-optic laryngoscope (8), two major problems are encountered, i.e. visualization of the glottis and the entry point at the level of vocal cords, and insertion of the fiberscope into the trachea (5,8).

Anesthesia, with or without the use of muscle relaxants, leads to paralysis of oropharyngeal muscles which in turn leads to a backward fall of the oropharyngeal structures such as the tongue and the epiglottis on the posterior wall of the pharynx (7,9). As a result, the space is compromised, with little of it available for passing the tube into the trachea (10,11). Some maneuvers such as jaw thrust, head flexion, tongue retraction, and use of intubator airways like Berman and Ovassapian have been successfully used to overcome the aforementioned problems. By pressing on the arytenoid cartilage, air passage can be occluded, as is in Selick's maneuver (2,6,9,12). Use of intubator laryngeal mask airway (ILMA) is another way to facilitate fiberscopic intubation (1,11,13). The goal of the NASAL-18 or the tube-first approach is to minimize the duration the fiberscope is between the cords, and also to reduce the time required to advance the tube into the larynx.

This study was designed to ascertain whether the facilitated method (NASAL-18) is superior to the conventional fiber-optic nasal intubation, as judged by the time for glottic visualization (T1) and the final time to intubation (T2).

## Methods

This study was carried out from January 2008 to June 2008 at the Poursina teaching hospital on 52 patients who were candidates for elective maxillofacial operation of type 1 LEFORTE fracture (miniplate insertion in the upper and lower jaws). Their ages ranged from 25 to 45 years, and they all fell within the American Society of Anesthesiologists (ASA) physical status classes of I and II. Patients were excluded if there was a contraindication for nasal intubation.

The patients were randomly allocated into two groups of nasal intubation using either the NASAL-18 or the conventional method (control) based on a table of randomization. In the control group, the fiberscope was inserted into the nasal cavity via the nostril and advanced through it till the vocal cords were visualized. Then, the nasal tube, which had been mounted on the scope beforehand, was glided over the scope and advanced through the vocal cords into the trachea. Whereas in the NASAL-18 group, or the tube-first approach, the nasal tube was inserted into the nasal cavity and advanced through it till the mark 18 reached the level of alae of the nose. With the tube advanced to the 18 cm at nare, the tube tip stands just above the larynx and breath sounds are audible through the tracheal tube. With additional anesthesia directed at the larynx (topical lidocaine), run the long scope through the tube, visualize the larynx, and pass between the cords towards the carina. The final depth of insertion of the nasal tube should be 26–28 cm at the nare, establishing its correct placement in the trachea as confirmed by bilateral audible breath sounds. Standard monitors including pulse oximetry, capnography, electrocardiography, and non-invasive blood pressure measurement were performed prior to the administration of intravenous medications and starting the nasal intubation. All patients received 2 microgram/kg of fentanyl, 3 mg of midazolam, and propofol at the rate of 30–35 μg/kg/min. Atropine (0.5 mg) was used to reduce mucus secretions of airways. After shrinkage of the nasal cavity by the instillation of phenylephrine drops (3 drops in each nostril), lidocaine spray (10%) was used to anesthetize the nasal and oral mucosae. With the use of 5 mL of 2% lidocaine transtracheally, the tracheal and bronchial mucosae were anesthetized. Nasal intubation with a spiral tube #7 followed. Three experienced anesthesiologists in fiber-optic nasal intubation were involved in fibroscopy of patients, and each anesthesiologist performed both techniques. The fiberscope used during this procedure was a Scholly type Flexilux 2000 with a length of 41 cm and a diameter of 3.7 mm. After dipping the fiberscope into the lubricating jelly, we inserted it into the nasal cavity until it reached the pharynx, and an attempt to locate the vocal cords then ensued.

During this period, patients were awake and co-operated well with the anesthesiologists. Time was recorded in seconds, from the start of insertion of fiber-optic laryngoscope in the nares till visualization of vocal cords (T1) and from this to successful intubation when the endotracheal tube cuff was inflated (T2). If it was necessary, facilitating techniques such as bronchoscopy, head flexion, and jaw thrusts were utilized. A time period of more than 180 s for the procedure or inability to intubate was considered as failed intubation, and the patient was then intubated using a different modality.

Statistical analysis was performed using the SPSS (SPSS Inc., Chicago, IL, USA) version 12. Student's *t* test was used to compare T1, T2, age, and weight, and chi-square test was used to compare ASA class, sex, and other categorical variables between groups. A *P* value < 0.05 was considered to be significant.

A linear regression analysis was utilized to compare T1 and T2 between control and NASAL-18 groups, controlling for the effect of age, sex, ASA class, and other variables.

### Ethical considerations

Approval of the study was obtained from the institutional ethical committee of human research, and written informed consent was obtained from each patient.

## Results

A total of 48 patients consented to participate in the study. There were no significant differences regarding age, sex, and ASA class between the two groups. As demonstrated (Table I) the T1 as well as total time of nasal intubation were significantly shorter in the NASAL-18 group than in the control group (*P* < 0.001), while there was no significant difference between the two groups regarding the T2 duration (*P* = 0.455).

**Table I.** Comparison of baseline characteristics, T1, T2, total time, and facilitation maneuvers between two groups, conventional (control) and NASAL-18.

Parameters	Conventional	NASAL-18	*P* value
Male sex (*n*)	22 (68.8%)	16 (66.7%)	0.86
ASA classes			
Class I	13 (40.6%)	5 (20.8%)	0.11
Class II	19 (59.4%)	19 (79.1%)	
Age (year)	34.5 ± 7.1	35.25 ± 7.9	0.71
Weight (kg)	73.9 ± 7.4	74.4 ± 9.0	0.84
T1 (s)	151.0 ± 56.5	65.2 ± 33.2	0.001
T2 (s)	25.1 ± 18.5	21.8 ± 10.2	0.45
Total time (s)	176.2 ± 56.3	87.1 ± 41.0	<0.001
Success (*n*)	16 (66.7%)	20 (83.3%)	0.18
Jaw thrust (*n*)	6 (25%)	6 (25%)	1
Head flexion (*n*)	8 (33.0%)	1 (4.2%)	0.02

Numerical data have been expressed as mean ± standard deviation. ASA = American Society of Anesthesiologists physical status classification; T1 = time from the start of insertion of the fiberscope into nares till visualization of vocal cord; T2 = time from here to complete intubation as T2.

Jaw thrust maneuver was used in 25% of the cases, which was the same in both groups (*P* > 0.05). Use of head flexion for facilitation of entry of the endotracheal tube was necessary in 4.2% and 33% of the cases in the NASAL-18 and control groups, respectively (*P* = 0.023). Success rates in the NASAL-18 and control groups were, respectively, 83% and 67%. In the NASAL-18 group, the failures were all related to the time it took us to intubate which exceeded the range specified in the ‘Methods’ section (mean 280 ± 30 s), whereas in the control group the failures were assignable to inability to intubate in 63% of the cases and prolonged intubation (T1+T2 > 180 s) in the remainders (mean 480 ± 20). This was higher than the corresponding time in the NASAL-18 group (*P* = 0.002). In the NASAL-18 and control groups, 7 and 14 cases, respectively, needed facilitating maneuvers such as jaw thrust and head flexion (*P* = 0.04).

Regression analysis indicated an inverse relationship between T1 and ASA class (*P* < 0.05). After adjusting for the effect of other variables, there was a significant difference between the two groups regarding T1 (Table II) but not T2.

**Table II.** Result of regression analysis for time from the start of insertion of the fiberscope into nares till visualization of vocal cord (T1) as the dependent variable has been summarized.

	Coefficient	*P* value
Model constant	33.6	–
Intervention	82.7	<0.001
Age (year)	1.5	0.11
Weight (kg)	−0.6	0.44
ASA classes	−30.8	0.04

Intervention shows the T1 change between two groups, conventional (control) and NASAL-18. ASA = American Society of Anesthesiologists physical status classification.

## Discussion

The incidence of difficulty in passing an endotracheal tube over an orally inserted fiberscope varies considerably between studies, ranging from 0% to 90% (5). Nasal fiber-optic intubation can be as difficult as oral fiber-optic intubation. Differences in the definition of the difficulty between various studies might have produced these differences in the reported incidences; however, other factors such as a different size of fiberscope, or else the type and size of the endotracheal tube, might also have affected the incidence. The major reason for difficulty in advancing an endotracheal tube over a fiberscope is considered to be deviation of the course of the tube from that of the fiberscope (because of the gap between the two) towards the epiglottis, arytenoid cartilage, pyriform fossae, or esophagus (12,14–16). Marfin et al. (5) showed that posterior structures of the laryngeal inlet are the sites of impingement during fiber-optic nasal intubation and suggested anti-clockwise rotation as its solution. Use of a spiral flexible tracheal tube, and aligning number 18 on the tube to the level of the nasal ala, had significantly improved the success rate of fibroscopy in our study. We chose number 18 because when this number is reached at the nasal alae, the endotracheal tube has sufficiently advanced to reach a point close to the vocal cords. In the NASAL-18 method, the tube acts as a tool that provides good alignment to guide the fiberscope through the nasal cavity to face the vocal cords. This may explain why T1 was shorter in the NASAL-18 group, though from this point there was no significant difference between endotracheal intubation times (T2) in the two groups. However, the NASAL-18 technique probably reduced fiberscope twisting as well as mucus secretions and bleeding as a consequence of milder traumatization of the upper airway tract that could be achieved due to a short T1, and all these put together helped to increase the success rate of nasal intubation.

Utilization of fiberscope without the use of any facilitating techniques has proven to be a difficult task. In this study we could see that the use of NASAL-18 technique allowed us to perform fibroscopy with greater ease and speed. The success rate for a quick and smooth intubation with the NASAL-18 method was about 83.3%, while this rate in the conventional method was at 67%. However, the power of this study to detect such a difference was only 16%. A large sample size is needed to compare the difference in more detail.

In conclusion, we can state that the NASAL-18 technique facilitates the success of fiber-optic nasal intubation in patients. The NASAL-18 method was found to be easier for the anesthesiologist and resulted in significantly faster glottic exposure. Although this technique needs to be explored by other researchers in larger studies in other craniomaxillofacial anomalies and other difficult cases of airway management, we believe that this technique has the potential ability to be added to the list of routine techniques of fiber-optic nasal intubation.
